# Supplementary material for: Complete Chloroplast Genome Sequences of Four Species in the Caladium Genus: Comparative and Phylogenetic Analyses
Source: Genes (Basel). 2022 Nov 22;13(12):2180. doi: 10.3390/genes13122180 (PMC9777821; doi:10.3390/genes13122180)
Supplement: Supplementary file 1 [file genes-13-02180-s001.zip › genes-2026138-supplementary/genes-2026138-supplementary/Supplementary Files/Figure S2.pdf]

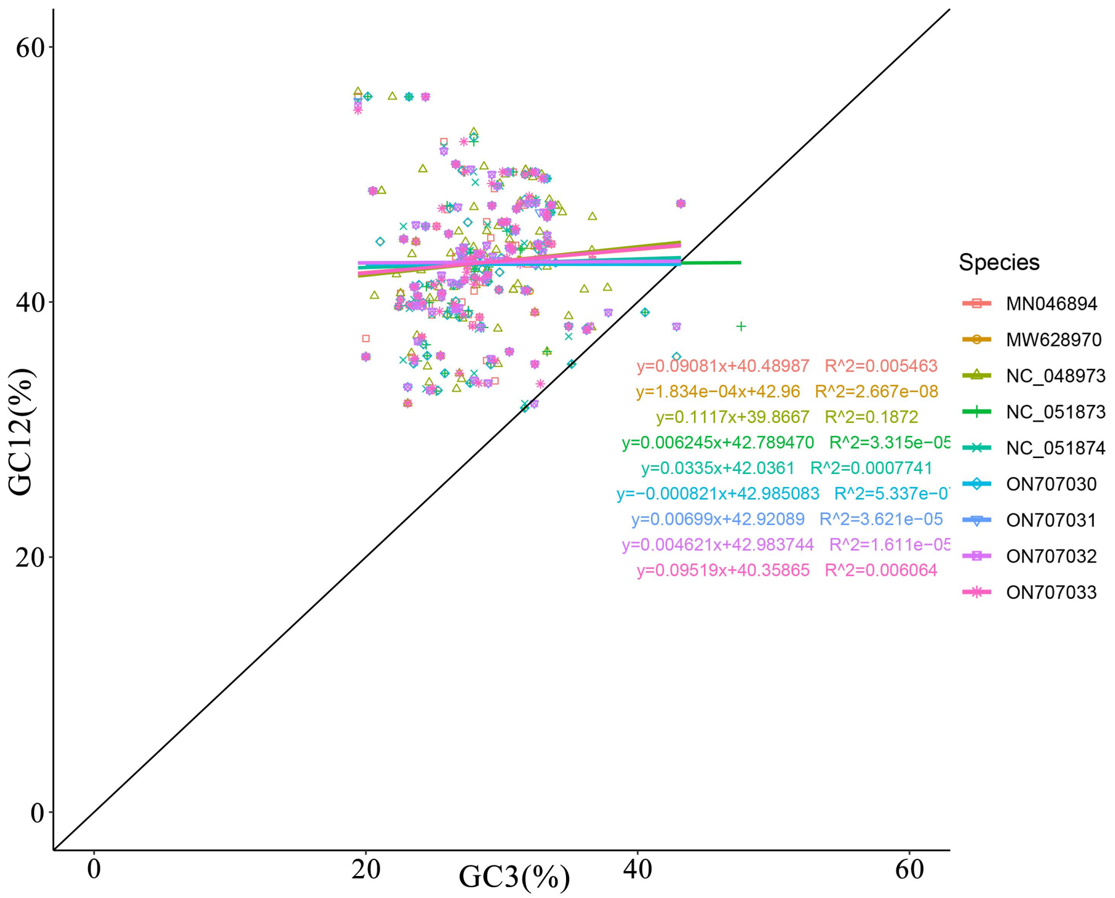

Figure S2. Neutrality analysis performed by plotting GC_{12} values against GC_3 values for the cp genomes of nine species of Araceae. The diagonal line on the neutrality plot shows that the value of GC_{12} is equal to GC_3.
