# Supplementary material for: Complete Chloroplast Genome Sequences of Four Species in the Caladium Genus: Comparative and Phylogenetic Analyses
Source: Genes (Basel). 2022 Nov 22;13(12):2180. doi: 10.3390/genes13122180 (PMC9777821; doi:10.3390/genes13122180)
Supplement: Supplementary file 1 [file genes-13-02180-s001.zip › genes-2026138-supplementary/genes-2026138-supplementary/Supplementary Files/Table S2.pdf]

Table S2. Functional classification of *Caladium* chlroplast genome.

Category	Code	Gene names	Amount
Subunit of Acetyl-CoA-carboxylase	*acc*	*accD*	1
Envelop membrane protein gene	*cem*	*cemA*	1
c-type cytochrom synthesis gene	*ccs*	*ccsA*	1
Protease gene	*clp*	*clpP1*	1
Maturase gene	*mat*	*matK*	1
ATP synthase	*atp*	*atpA, atpB, atpE, atpF, atpH, atpI*	6
NADH dehydrogenase	*ndh*	*ndhA, ndhB* (×2), *ndhC, ndhD, ndhE, ndhF, ndhG, ndhH, ndhI, ndhJ, ndhK*	12
Cytochrome b/f complex	*pet*	*petA, petB, petD, petG, petL, petN*	6
Photosystem I	*psa*	*psaA, psaB, psaC, psaI, psaJ*	5
Photosystem II	*psb*	*psbA, psbB, psbC, psbD, psbE, psbF, psbH, psbI, psbJ, psbK, psbL, psbM, psbT, psbZ*	14
Rubisco	*rbc*	*rbcL*	1
Large subunit ribosomal proteins	*rpl*	*rpl14, rpl16, rpl2* (×2), *rpl20, rpl22, rpl23* (×2), *rpl32, rpl33, rpl36*	11
RNA polymerase	*rpo*	*rpoA, rpoB, rpoC1, rpoC2*	4
Small subunit ribosomal proteins	*rps*	*rps11, rps12* (×2), *rps14, rps15, rps16, rps18, rps19, rps2, rps3, rps4, rps7* (×2), *rps8*	14
Ribosomal RNAs	*rrn*	*rrn16* (×2), *rrn23* (×2), *rrn4.5* (×2), *rrn5* (×2),	8
Transfer RNAs	*trn*	*trnA-UGC* (×2), *trnC-GCA, trnD-GUC, trnE-UUC, trnF-GAA, trnG-GCC, trnG-UCC, trnH-GUG, trnI-CAU* (×2), *trnI-GAU* (×2), *trnK-UUU, trnL-CAA* (×2), *trnL-UAA, trnL-UAG, trnM-CAU, trnN-GUU* (×2), *trnP-UGG, trnQ-UUG, trnR-ACG* (×2), *trnR-UCU, trnS-GCU, trnS-GGA, trnS-UGA, trnT-GGU, trnT-UGU, trnV-GAC* (×2), *trnV-UAC, trnW-CCA, trnY-GUA, trnfM-CAU*	37
Hypothetical chloroplast reading frames	*ycf*	*ycf1* (×2), *ycf2* (×2)	4
Other proteins		*pafI, pafII, pbf1, infA*	4

Note: (×2) indicates the number of gene duplication.
