# Supplementary material for: Complete Chloroplast Genome Sequences of Four Species in the Caladium Genus: Comparative and Phylogenetic Analyses
Source: Genes (Basel). 2022 Nov 22;13(12):2180. doi: 10.3390/genes13122180 (PMC9777821; doi:10.3390/genes13122180)
Supplement: Supplementary file 1 [file genes-13-02180-s001.zip › genes-2026138-supplementary/genes-2026138-supplementary/Supplementary Files/Table S3.pdf]

Table S3. Determination of optimal codons in the chloroplast genomes of *Caladium*.

Codon	Amino acid	Number				Relative synonymous codon usage			
		C. bicolor	***C. humboldtii***	***C. praetermissum***	***C. lindenii***	***C. bicolor***	***C. humboldtii***	***C. praetermissum***	***C. lindenii***
GCA	Ala	414	414	415	419	1.184	1.184	1.181	1.191
GCC	Ala	217	217	219	219	0.620	0.620	0.623	0.623
GCG	Ala	145	145	143	143	0.415	0.415	0.407	0.407
GCU	Ala	623	623	628	626	1.781	1.781	1.788	1.780
AGA	Arg	523	523	515	524	1.956	1.958	1.940	1.953
AGG	Arg	154	154	156	156	0.576	0.576	0.588	0.581
CGA	Arg	356	356	354	357	1.332	1.333	1.333	1.330
CGC	Arg	102	102	103	102	0.382	0.382	0.388	0.380
CGG	Arg	110	110	113	112	0.411	0.412	0.426	0.417
CGU	Arg	359	358	352	359	1.343	1.340	1.326	1.338
AAC	Asn	295	296	298	296	0.470	0.472	0.472	0.468
AAU	Asn	960	959	966	970	1.530	1.528	1.528	1.532
GAC	Asp	214	214	217	217	0.399	0.399	0.402	0.399
GAU	Asp	859	859	862	870	1.601	1.601	1.598	1.601
UGC	Cys	71	71	76	75	0.473	0.473	0.502	0.493
UGU	Cys	229	229	227	229	1.527	1.527	1.498	1.507
CAA	Gln	699	698	697	698	1.495	1.495	1.496	1.491
CAG	Gln	236	236	235	238	0.505	0.505	0.504	0.509
GAA	Glu	1025	1025	1035	1045	1.480	1.480	1.491	1.486
GAG	Glu	360	360	353	361	0.520	0.520	0.509	0.514
GGA	Gly	727	727	730	728	1.647	1.647	1.640	1.638
GGC	Gly	167	166	166	165	0.378	0.376	0.373	0.371
GGG	Gly	296	297	307	300	0.670	0.673	0.690	0.675
GGU	Gly	576	576	578	585	1.305	1.305	1.298	1.316
CAC	His	154	154	156	158	0.488	0.487	0.491	0.496
CAU	His	477	478	480	479	1.512	1.513	1.509	1.504
AUA	Ile	701	701	706	706	0.945	0.945	0.945	0.941
AUC	Ile	425	425	426	431	0.573	0.573	0.570	0.575
AUU	Ile	1100	1100	1110	1113	1.482	1.482	1.485	1.484
CUA	Leu	367	368	368	370	0.812	0.814	0.813	0.817
CUC	Leu	179	179	181	181	0.396	0.396	0.400	0.400
CUG	Leu	166	166	162	166	0.367	0.367	0.358	0.366
CUU	Leu	586	585	580	583	1.296	1.294	1.281	1.287
UUA	Leu	835	835	837	832	1.847	1.847	1.848	1.837
UUG	Leu	579	580	589	586	1.281	1.283	1.301	1.294
AAA	Lys	1033	1034	1058	1041	1.483	1.484	1.479	1.477
AAG	Lys	360	360	373	369	0.517	0.516	0.521	0.523

AUG	Met	608	608	608	602	1.997	1.997	1.997	1.997
GUG	Met	1	1	1	1	0.003	0.003	0.003	0.003
UUC	Phe	537	537	540	540	0.714	0.714	0.715	0.718
UUU	Phe	968	967	971	965	1.286	1.286	1.285	1.282
CCA	Pro	330	330	331	335	1.220	1.220	1.211	1.230
CCC	Pro	205	205	206	202	0.758	0.758	0.754	0.742
CCG	Pro	117	117	119	118	0.433	0.433	0.435	0.433
CCU	Pro	430	430	437	434	1.590	1.590	1.599	1.594
AGC	Ser	104	104	102	104	0.303	0.303	0.294	0.299
AGU	Ser	427	427	441	441	1.245	1.245	1.272	1.267
UCA	Ser	435	435	436	443	1.268	1.268	1.257	1.273
UCC	Ser	342	342	344	344	0.997	0.997	0.992	0.989
UCG	Ser	178	178	176	180	0.519	0.519	0.507	0.517
UCU	Ser	572	572	582	576	1.668	1.668	1.678	1.655
UAA	Ter	41	41	40	40	1.430	1.430	1.395	1.395
UAG	Ter	26	26	26	27	0.907	0.907	0.907	0.942
UGA	Ter	19	19	20	19	0.663	0.663	0.698	0.663
ACA	Thr	436	436	435	437	1.290	1.290	1.278	1.282
ACC	Thr	243	243	246	245	0.719	0.719	0.723	0.718
ACG	Thr	151	151	152	152	0.447	0.447	0.447	0.446
ACU	Thr	522	522	528	530	1.544	1.544	1.552	1.554
UGG	Trp	452	452	451	452	1.000	1.000	1.000	1.000
UAC	Tyr	204	204	204	206	0.420	0.420	0.421	0.421
UAU	Tyr	768	768	764	773	1.580	1.580	1.579	1.579
GUA	Val	518	518	521	526	1.425	1.425	1.426	1.438
GUC	Val	187	187	190	189	0.514	0.514	0.520	0.517
GUG	Val	206	206	209	209	0.567	0.567	0.572	0.571
GUU	Val	543	543	541	539	1.494	1.494	1.481	1.474
