# Supplementary figures and images for: Complete Chloroplast Genome Sequences of Four Species in the Caladium Genus: Comparative and Phylogenetic Analyses
Source: Genes (Basel). 2022 Nov 22;13(12):2180. doi: 10.3390/genes13122180 (PMC9777821; doi:10.3390/genes13122180)

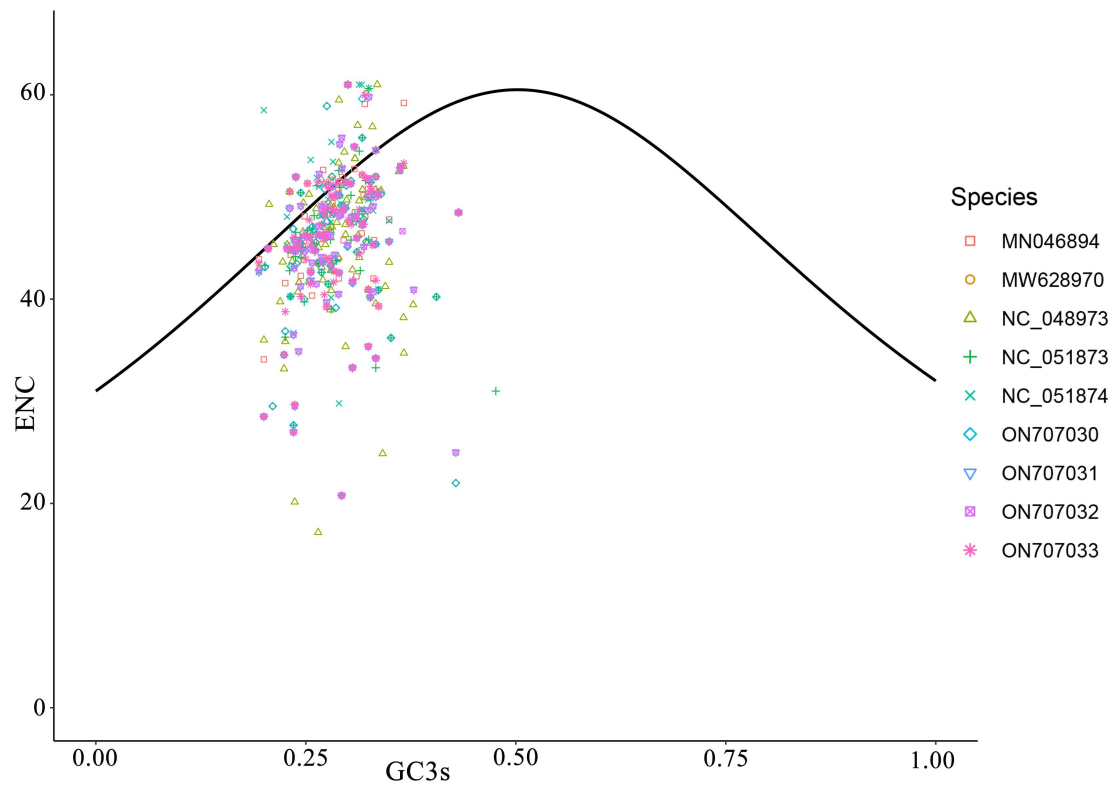

**Figure S1.** Analysis of ENC-plot in the cp genomes of nine species of Araceae.

Supplement: Supplementary file 1 [file genes-13-02180-s001.zip › genes-2026138-supplementary/genes-2026138-supplementary/Supplementary Files/Figure S1.pdf]
